# Identification of AP-1 as a Critical Regulator of Glutathione Peroxidase 4 (GPX4) Transcriptional Suppression and Acinar Cell Ferroptosis in Acute Pancreatitis

**DOI:** 10.3390/antiox12010100

**Published:** 2022-12-31

**Authors:** Xiaojie Ma, Xiaowu Dong, Yao Xu, Nan Ma, Mei Wei, Xiaochun Xie, Yingying Lu, Wangsen Cao, Guotao Lu, Weiqin Li

**Affiliations:** 1 Department of Critical Care Medicine, Affiliated Jinling Hospital, Medical School of Nanjing University, Nanjing 210016, China; 2 Department of Critical Care Medicine, Jinling Hospital, Nanjing Medical University, Nanjing 210016, China; 3 Department of Critical Care Medicine, Jinling Hospital, Medical School of Southeast University, Nanjing 210016, China; 4 Pancreatic Center, Department of Gastroenterology, Yangzhou Key Laboratory of Pancreatic Disease, Affiliated Hospital of Yangzhou University, Yangzhou University, Yangzhou 225000, China

**Keywords:** acute pancreatitis, ferroptosis, active protein 1, transcription factor, GPX4

## Abstract

Glutathione peroxidase 4 (GPX4)-dependent ferroptosis in pancreatic acinar cells plays a critical role in acute pancreatitis (AP). However, potential upstream regulators of GPX4 are not well defined. Here, we observed a marked reduction in acinar GPX4 expression and ferroptotic cell death in mice with cerulein-induced AP. To determine the critical factors involved in acinar cell ferroptosis, pancreas transcriptome data from an AP mouse model were analyzed and overlapped with predicted transcription factors of *Gpx4*, and an upregulated transcription factor active protein 1 (AP-1) protein, Jun, was identified. The administration of a specific ferroptosis inhibitor liproxstatin-1 alleviated AP pathology and significantly decreased Jun levels. Bioinformatic analysis indicated that the *Gpx4* promoter contains a putative AP-1 binding site. Jun binds directly to the *Gpx4* promoter and inhibits *Gpx4* transcription under pancreatic conditions. AP-1 inhibition by a selective inhibitor SR11302 reversed GPX4 reduction and ameliorated AP pathology in a GPX4-dependent manner. Collectively, our study demonstrates that the downregulation of GPX4 by AP-1 is critical in the aggravation of acinar cell ferroptosis during the progression of AP. Strategies targeting the AP-1/GPX4 axis may be potentially effective for the prevention and treatment of AP.

## 1. Introduction

Acute pancreatitis (AP) is an acute inflammatory self-digestive disease of the exocrine pancreas. A variety of damaging factors (gallstones, high triglycerides, alcohol, etc.) induce trypsin activation in acinar cells, which results in acinar cell injury and early local inflammation in the pancreas [1,2]. Most patients with AP self-recover after conservative treatment; however, about one in five patients develop severe acute pancreatitis (SAP) with excessive systemic inflammation and multiple organ failure, resulting in prolonged hospital stay and high mortality [3,4,5]. The main treatments for AP include aggressive intravenous hydration, early enteral feeding, and intervention in the event of complication; however, there are no causal treatments and the therapies are based on symptomatic treatment [6]. Thus, exploring the precise mechanism and identifying key regulators of AP will provide new insights into the therapeutic strategies for AP treatment.

Redox signaling and oxidative stress play critical roles in local and systemic inflammatory responses during AP [7]. Among the factors that cause oxidative stress in cells, lipid peroxidative modification of membrane bilayer species is an important regulator of cell fate, and extensive lipid peroxide accumulation results in cell death via an iron-dependent paradigm termed ferroptosis [8,9,10]. There are various forms of programmed cell death identified so far, including apoptosis, necroptosis, pyroptosis, ferroptosis, and entosis, as well as some newly reported cell death forms such as methuosis and parthanatos [11]. A recent study suggested that high extracellular trypsin increases the pancreatic acinar cells’ susceptibility to ferroptosis rather than apoptosis and necroptosis [12]. Ferroptotic cell death can be suppressed by many inhibitors, such as Liproxstatin-1 (Lip-1), which specifically inhibits ferroptosis via prevention of lipid peroxidation [13,14]. Glutathione peroxidase 4 (GPX4) serves as a key regulator of ferroptosis and scavenges lipid peroxides, whereas inactivation of GPX4 leads to oxidation imbalance, membrane structure dysfunction, and ferroptosis [15]. The pathogenesis of AP is associated with various forms of regulated cell death, including pyroptosis, necroptosis and ferroptosis [12,16,17]. Unlike the other forms of regulated cell death, ferroptosis is characterized by lipid peroxidation mainly due to glutathione reduction, GPX4 inactivation and lipotoxicity [13]. It is believed that ferroptosis exacerbates pancreatic injury and systemic inflammatory responses during AP [18]. Recent studies have demonstrated that iron metabolism imbalance and GPX4-dependent ferroptosis play important roles in AP progression; however, the upstream molecules and specific mechanisms that regulate GPX4 and subsequent ferroptosis in AP remain elusive [12,18,19,20,21,22].

Here, we investigated the role of active protein 1 (AP-1), a pro-inflammatory factor, in regulating GPX4-dependent ferroptosis and AP. AP-1 was screened out from pancreas transcriptomic data and was identified to be involved in the transcriptional control of GPX4. To determine the role of AP-1 in ferroptosis-related AP, we used a selective AP-1 inhibitor SR11302 in both in vitro and in vivo experiments [23]. We demonstrated that AP-1 suppressed GPX4 transcription via binding to the promoter region of GPX4, which aggravates ferroptosis and finally increases the severity of AP. Taken together, our study uncovers the functionality of AP-1 and GPX4 in the pathogenesis of ferroptosis as well as their interplay in the regulation of AP.

## 2. Materials and Methods

### 2.1. Experimental AP Animal Models and Drug Administration

A total of 144 male wild-type (WT) C57BL/6 mice, 48 conditional *Gpx4* knockout mice and 48 *Gpx4*^fl/fl^ mice were used for experiments in this study. Male wild-type (WT) C57BL/6 mice (6–8 weeks old) were purchased from GemPharmatech Co. Ltd. (Nanjing, Jiangsu, China). The conditional *Gpx4* knockout mice (*Ptf1a*^Cre^*Gpx4*^fl/fl^, *Gpx4*KO) were generated by crossing *Gpx4*^fl/fl^ mice (JAX stock #027964) with *Ptf1a*^Cre^ mice (also named *Ptf1a*^Cre-ERTM^, JAX stock #019378). Both strains were purchased from Jackson Laboratory (Bar Harbor, ME, USA) and were of C57BL/6 background. The *Ptf1a*^Cre-ERTM^ mice express a Cre-ERTM fusion protein (ERTM is a mutated version of the ligand binding domain of estrogen receptor) under the transcriptional control of the pancreas specific transcription factor 1α (*Ptf1a*) promoter and its activation is induced by intraperitoneal injection of tamoxifen (75 mg/kg per mouse dissolved in corn oil, daily injection for five days and administered for two weeks before experiments). The mice were genotyped by PCR analysis of tail tip DNA and *Gpx4* deletion was verified by western blotting. *Gpx4*^fl/fl^ mice injected with tamoxifen served as controls. To induce AP, cerulein (CER) (Pep03263, Nanjing Peptide, Nanjing, China) was intraperitoneally and daily injected (200 μg/kg dissolved in 100 μL phosphate buffered saline, 1 h interval, 10 times), while the control group received an equal volume of phosphate buffered saline. WT mice were randomly divided into three groups: 1, Controls (Ctrl); 2, CER and 3, CER+liproxstatin-1 (Lip-1, HY-12726, MedChemExpress, USA, 10 mg/kg in 100 μL containing 10% dimethyl sulfoxide and 18% sulfobutylether-β-cyclodextrin in saline, intraperitoneally injected twice, immediately and 6 h after the first injection of CER) or CER+SR11302 (HY-15870, 1 mg/kg, one intraperitoneal injection of 100 μL dissolved in the same solvent as Lip-1). For the *Gpx4*KO mouse assay, *Gpx4*^fl/fl^ and *Gpx4*KO mice were injected CER with or without SR11302 as above. WT C57BL/6 mice (6 mice per group) or *Gpx4*KO mice (6 mice per group) were randomly assigned to each group for subsequent experiments. After AP induction, mice were sacrificed 12 h later. All mice were euthanized by intraperitoneal injection of pentobarbital sodium. The use of animals and all experimental protocols were approved by the Experimental Animal Ethics Committee of Affiliated Jinling Hospital, Medical School of Nanjing University (No. 2021DZGKJDWLS-0057, annually renewable) in accordance with the National Institutes of Health Guide for the Care and Use of Laboratory Animals (NIH Publications No. 8023, revised 1978).

### 2.2. Microarray Data GEO Database Collection and Analysis

Relevant Microarray data of AP in mice were obtained from the NCBI GEO database (GSE109227) [24], which was generated from the Affymetrix Mouse Gene 1.0 ST Array, including 6 AP mice injected with cerulein and 5 control mice injected with sodium chloride. The basic analysis was performed with the Genespring software (version 14.8, Agilent Technologies), in which the raw data was normalized based on the quantile algorithm. The probes with at least 75% samples in one group flagged “Detected” were selected for further data analysis. The differentially-expressed genes were identified based on fold changes and *p* values calculated by Student’s *t*-test. The thresholds for up- and down-regulated genes were set as fold change ≥ 2.0 and *p* value ≤ 0.05. Kyoto Encyclopedia of Genes and Genomes (KEGG) pathway enrichment analysis of differentially-expressed genes was performed using R to screen the significant enriched term [25].

### 2.3. Amylase and Lipase Analysis

Orbital venous blood samples were obtained for amylase and lipase assay. The blood samples were centrifuged at 4000 rpm for 10 min at 4 °C, and the upper sera were collected and stored at −80 °C for further analysis. Serum amylase (100000060, Biosino Bio-technology, Beijing, China) and lipase (A054-2, Nanjing Jiancheng Bioengineering Institute, Nanjing, China) were analyzed using the respective kits according to the manufacturer’s protocols.

### 2.4. Histopathology and Immunohistochemistry Analysis

The pancreatic tissues were stained with hematoxylin and eosin (H&E). Histopathological damage was assessed based on the severity of edema, inflammatory cell infiltration, and acinar cell necrosis by two independent pathologists in a blinded manner. The histopathological scoring for pancreatic injury was based on three parameters of a 0–4/5 points scoring standard [26]. The severity of edema was assessed as follows: 0, absent; 1, diffuse expansion of interlobar septae; 2, diffuse expansion of interlobubar septae; 3, diffuse expansion of interacinar septae; and 4, diffuse expansion of intercellular spaces. For assessing acinar cell necrosis, 0 represented no necrotic cell, while 1, 2, 3, 4 represented 1–4, 5–10, 11–16, >16 necrotic cells in each high-power field, respectively. For inflammation, 0 represented 0–5 leukocytes in each high-power field, while 1, 2, 3, 4 represented 6–15, 16–25, 26–35, and >35 leukocytes in each high-power field, respectively. Acinar cell necrosis and inflammatory infiltration were counted as the average number per 10 high power fields at magnification 400×. For immunohistochemistry, slides of pancreatic tissue were treated with dewaxing, hydration, antigen retrieval, blocking endogenous peroxidase, and blocking with normal goat serum. Thereafter, slides were incubated overnight with anti-4-hydroxynonenal (4-HNE) antibody (ab46545, 1:100 dilution, Abcam), anti-GPX4 antibody (ab125066, 1:500 dilution, Abcam, Cambridge, MA, USA), and anti-Jun antibody (9165, 1:500 dilution, Cell Signaling, Danvers, MA, USA) at 4 °C, followed by incubation with biotinylated secondary antibody (SP-9000, OriGene, Rockville, MD, USA) at room temperature for 15 min. Afterwards, an appropriate amount of freshly prepared DAB chromogenic solution (AR1027, BOSTER, Wuhan, China) was added to process the slides, and the nuclei were counterstained with hematoxylin. A light microscope was used to capture images (IX73, OLYMPUS, Tokyo, Japan).

### 2.5. Cell Culture and Transfection

Mouse pancreatic acinar carcinoma 266-6 cells (CRL-2151, ATCC, Manassas, VA, USA) were cultured in Dulbecco’s modified Eagle’s medium supplemented with 10% foetal bovine serum, 100 U/mL penicillin, and 100 μg/mL streptomycin (15140122, Thermo Fisher, Waltham, MA, USA) in humidified atmosphere of 5% CO_2_ incubator at 37 °C.

In accordance with the manufacturer’s protocol, cells were transfected with GPX4 siRNA or Jun siRNA for 36 h using Lipofectamine 3000 (L3000015, Invitrogen, Carlsbad, CA, USA), whereas the control group was transfected with siCONTROL as a negative control. RSL3 (200 nM, HY-100218A, MedChemExpress, Monmouth Junction, NJ, USA) or Cholecystokinin (CCK) (5 μM, HY-P0093, MedChemExpress, Monmouth Junction, NJ, USA) was treated with transfected cells for 6 h following transfection. Western blot analysis was used to evaluate the knockdown efficacy. The siRNA sequences were as follows:

siGpx4 (NCBI Gene ID: 625249), 5ʹ-CCAGGAAGUAAUCAAGAAATT-3ʹ,

siJun (NCBI GeneID: 16476), 5ʹ-GGCACAGCUUAAGCAGAAATT-3ʹ.

### 2.6. Quantitative Real-Time PCR Analysis

Following the manufacturer’s instructions, TRIzol (15596018, Invitrogen, Carlsbad, CA, USA) was used to extract the total RNA from pancreatic tissue or 266-6 cells. RNA was reverse transcribed to cDNA using a reverse transcriptase kit (RR036A; TaKaRa, Tokyo, Japan). The resulting cDNA was used for real-time qPCR with SYBR Green PCR Master Mix (11201ES03, Yeasen, Shanghai, China) following the manufacturer’s protocols. The internal reference gene is *Gapdh* (NCBI Gene ID: 14433). The abundance of *Gpx4* (NCBI Gene ID: 625249) mRNAs were determined by relative expression to the respective *Gapdh* by the 2^−ΔΔCt^ method [27]. The following primer sequences were utilized:

*Gpx4* F: 5ʹ-GCCTGGATAAGTACAGGGGTT-3ʹ,

*Gpx4* R: 5ʹ-CATGCAGATCGACTAGCTGAG-3ʹ,

*Gapdh* F: 5ʹ-AGGTCGGTGTGAACGGATTTG-3ʹ,

*Gapdh* R: 5ʹ-TGTAGACCATGTAGTTGAGGTCA-3ʹ.

### 2.7. Chromatin Immunoprecipitation

Using the SimpleChIP enzymatic Chromatin IP kit with magnetic beads (9005, Cell Signaling, Danvers, MA, USA) in accordance with the manufacturer’s instructions, chromatin immunoprecipitation (ChIP) was performed using 266-6 cells to detect the association of Jun with the *Gpx4* promoter under various conditions. Immunoprecipitation was performed with an anti-Jun antibody (9165, Cell Signaling Technology, Danvers, MA, USA) or an isoform-matched IgG as the control. The *Gpx4* promoter-specific PCR primers were used to examine the immunoprecipitated DNA. The *Gpx4* (NCBI Gene ID: 625249) promoters are as follows:

primer1 F: 5ʹ-AGGGATTAGAGTCCAGGCGA-3ʹ,

primer1 R: 5ʹ- CCCAACAGTTCCTCCTGCAA-3ʹ,

primer2 F: 5ʹ-ATTAGAGTCCAGGCGAGGGC-3ʹ,

primer2 R: 5ʹ-TTCCTCCTGCAACTTCACC-3ʹ.

Thereafter, the percent input was calculated to measure GPX4 expression as a percentage of the global input chromatin.

### 2.8. Western Blotting

Western blotting analysis was performed on the pancreatic tissue. The primary antibodies used for immunoblotting were anti-GPX4 antibody (ab125066, 1:1000 dilution, Abcam, Cambridge, MA, USA), anti-c-Jun antibody (9165, 1:1000 dilution, Cell Signaling, Danvers, MA, USA) and anti-GAPDH antibody (ab8245, 1:5000 dilution, Abcam, Cambridge, MA, USA). WB was analyzed by a chemiluminescence imaging system (Tanon, China). The densitometry of the target proteins was normalized to the loading control GAPDH and presented as fold changes relative to the control.

### 2.9. Cell Lipid Reactive Oxygen Species (ROS) Assay

The 266-6 cells were seeded in 48-well plates at 1 × 10^5^ cells per well, treated as designed, and subsequently stained with 2 μM C11-BODIPY (D3861, Invitrogen, Carlsbad, CA, USA) for 30 min in the dark. Cells were extracted using trypsinization after being washed with phosphate-buffered saline (PBS), and were then examined using a flow cytometer.

### 2.10. Dual-Luciferase Assay

The pGL3 basic luciferase vector was used to clone the GPX4 promoter (NM 001367995), as well as mutant (Jun-mut) and deletion mutant (Luc1 to Luc3, Luc1 as wild-type promoter) GPX4 promoter sequences. Jun coding sequences were synthesized and cloned into pcDNA3.1. The sequence accuracy of the inserts was confirmed via Sanger sequencing. HEK293T cells were transfected with the Jun plasmid and different GPX4 promoters plus Renilla plasmid for transfection control, using Lipofectamine 3000. To measure luciferase activity, a dual-luciferase reporter assay system (E2920, Promega Corporation, Madison, WI, USA) was used.

### 2.11. Cytotoxicity Assay

The release of lactic dehydrogenase (LDH) into the cells was measured using the LDH Cytotoxicity Assay kit (CK12, Dojindo, Kumamoto, Japan) to assess cell death.

### 2.12. Statistical Analysis

Data were analyzed using the GraphPad Prism software (version 5.0; GraphPad, San Diego, CA, USA) and are presented as the mean ± standard error of mean. Parametric tests (Student’s *t*-test for comparison between two groups, or one-way ANOVA for three or more groups followed by Tukey’s test) were carried out using continuous data. Statistical significance was defined as a two-tailed *p* value less than 0.05. *, *p* < 0.05; **, *p* < 0.01; ***, *p* < 0.001; ns, not significant.

## 3. Results

### 3.1. AP Progression was Associated with GPX4-Dependent Ferroptosis and Jun Elevation

**Figure 1:**
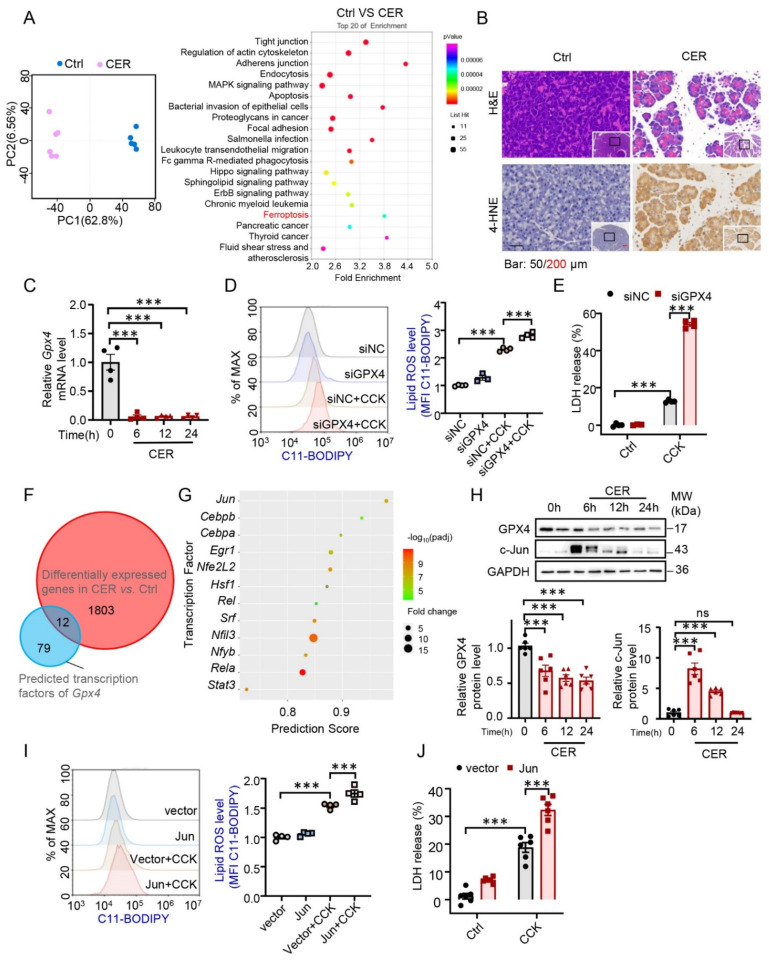
Acute pancreatitis (AP) progression was associated with glutathione peroxidase 4 (GPX4)-dependent ferroptosis and Jun elevation. (**A**) The principal component analysis and the enrichment analysis of Kyoto Encyclopedia of Genes and Genomes signaling pathways in cerulein (CER)-induced AP group compared with the control group from pancreas transcriptome data (GSE109227). (**B**) Representative photomicrographs of histopathology and 4-hydroxynonenal (4-HNE) using immunohistochemistry (IHC) in the pancreas of mice (control [Ctrl] VS CER, *n* = 4). (**C**) The mRNA of GPX4 in the pancreas of CER-induced AP mice were measured using qPCR (*n* = 4). (**D**,**E**) After transfection with GPX4 siRNA, the cells were treated with cholecystokinin (CCK) (5 μM) for 6 h, and lipid reactive oxygen species (ROS) levels and the extent of cell death were determined (*n* = 4). (**F**) Differently expressed genes in pancreas transcriptome analysis were overlapped with predicted transcription factors of GPX4. (**G**) Fold change and prediction score of selected transcription factors in CER group compared with control group. (**H**) The protein expression of GPX4 and Jun in the pancreas of CER-induced AP mice were measured using western blotting (*n* = 6). (**I**,**J**) After transfected with plasmid of Jun for 36 h, the cells were treated with CCK (5 μM) for 6 h, and lipid ROS levels and the extent of cell death were determined (*n* = 4–6). ***, *p* < 0.001; ns, not significant.

To identify the critical factors implicated in AP, we first examined gene expression profiles in pancreatic transcriptome data from mice with CER-induced AP and controls (GSE109227). The repeatability of biological replicates and the separation between AP and control were confirmed by the principal component analysis. The KEGG analysis revealed that differentially expressed genes (DEGs) were enriched in the ferroptosis pathway in CER-induced AP (Figure 1A). Consistent with the above findings in RNA sequence analysis, we observed pancreatic injury and elevated levels of 4-HNE after AP, which is regarded as a typical lipid peroxide during ferroptotic cell death (Figure 1B). GPX4 inactivation leads to lipid peroxide accumulation and subsequent ferroptosis. Thus, we determined the expression of GPX4 during AP. As expected, both the mRNA and protein levels of GPX4 were downregulated after intraperitoneal injection of CER (Figure 1C,H). Thereafter, we determined whether impaired expression of GPX4 was responsible for the aggravation of CCK-induced AP in vitro. Knockdown of GPX4 significantly increased the cellular lipid peroxidation level and resulted in an increase in LDH release (Figure 1D,E).

Thereafter, DEGs in pancreatic transcriptome analysis overlapped with the predicted transcription factors of GPX4 (https://tfbind.hgc.jp/). Twelve transcription factors were screened, all of which were upregulated (Figure 1F). We found that *Jun* bound to the GPX4 promoter region with the greatest probability (Figure 1G). We observed that the protein level of Jun dramatically increased in mice with CER-induced AP (Figure 1H). Thereafter, we tested AP-1-mediated oxidant response in vitro; as expected, the overexpression of Jun significantly aggravated the CCK-induced lipid ROS level (Figure 1I) and LDH release (Figure 1J). Collectively, these results revealed a close association between AP progression, ferroptosis, and Jun elevation.

### 3.2. Inhibition of Ferroptosis Alleviated Experimental AP and Decreased Jun Level

**Figure 2:**
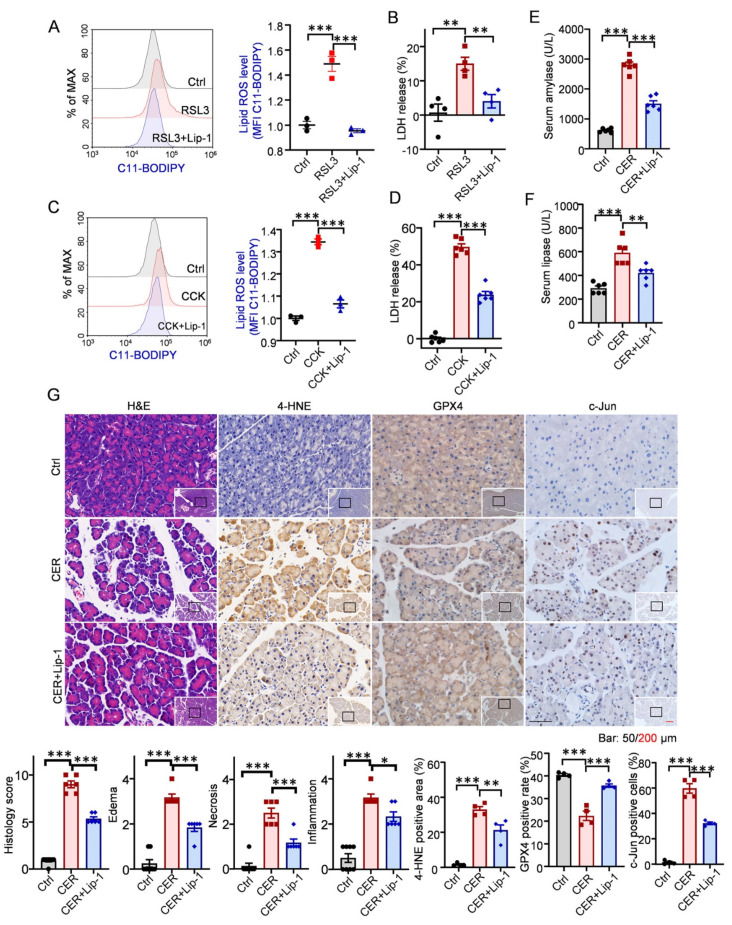
Blocking ferroptosis alleviated the experimental acute pancreatitis and inhibited Jun. (**A**–**D**) The 266-6 cells were treated with RSL3 (200 nM) or cholecystokinin (CCK) (5 μM) with or without liproxstatin-1 (Lip-1, 10 μM, ferroptosis inhibitor) for 6 h; lipid reactive oxygen species (ROS) levels and extent of cell death were determined (*n* = 4–6). (**E**,**F**) Serum amylase and lipase levels (*n* = 6). (**G**) Representative hematoxylin and eosin (H, E) staining of pancreatic tissues in magnifications 100× and 400× (*n* = 6). Representative photomicrographs of pancreas of 4-hydroxynonenal (4-HNE), Jun, and glutathione peroxidase 4 (GPX4) via immunohistochemistry (*n* = 4). *, *p* < 0.05; **, *p* < 0.01; ***, *p* < 0.001; ns, not significant.

Lip-1 was used as a potent and specific ferroptosis inhibitor [13]. We found that Lip-1 alleviated both lipid ROS and LDH levels in RSL3-treated 266-6 cells (Figure 2A,B). Similarly, we observed that Lip-1 decreased levels of lipid ROS and LDH in CCK-treated acinar cells (Figure 2C,D), indicating that CCK-induced ferroptotic cell death was inhibited by Lip-1. Thereafter, we determined the impact of ferroptosis on CER-induced AP in mice. Consistent with the in vitro results, Lip-1 treatment significantly reduced the serum amylase and lipase levels in experimental AP (Figure 2E,F). Examination of pancreatic tissues from Lip-1-treated mice revealed alleviated histological injury, manifested as decreased edema, inflammatory cell infiltration, and acinar necrosis (Figure 2G). Thereafter, immunohistochemistry staining assays were performed, and it was found that the expression of GPX4 was elevated, whereas that of 4-HNE and Jun was inhibited by Lip-1 (Figure 2G). These data collectively demonstrated that blocking ferroptosis decreases the severity of AP and suggested that AP-1 may be a potential target to mediate ferroptosis and AP.

### 3.3. Jun Inhibited Gpx4 Transcription

**Figure 3:**
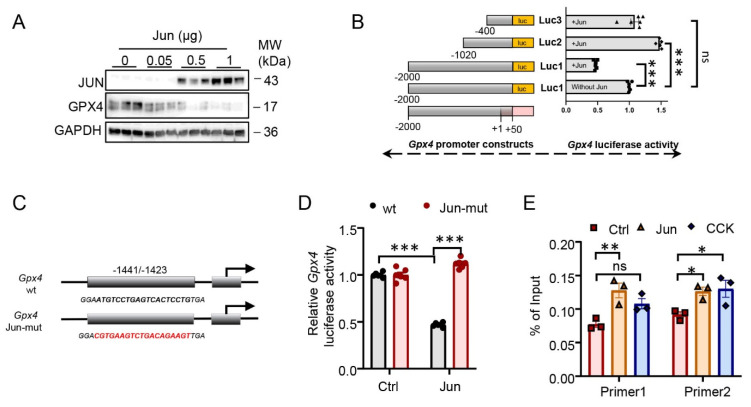
Jun inhibited glutathione peroxidase 4 (*Gpx4*) transcription. (**A**) The protein level of Jun or GPX4 in 266-6 cell transfected with Jun-overexpressing plasmid in 266-6 cells (*n* = 3). (**B**) Luciferase reporter assays of *Gpx4* promoter fragments fused with a luciferase reporter gene (Luc1 to Luc3) in Jun-transfected HEK293T (*n* = 6). (**C**) A mutation of the Jun binding site on the *Gpx4* promoter (−1441–−1423) was constructed (*Gpx4* Jun-mut). (**D**) Reporter activity of wild-type *Gpx4* (*Gpx4* wt) or mutated *Gpx4* (*Gpx4* Jun-mut) with Jun overexpression (*n* = 6). (**E**) Chromatin immunoprecipitation (ChIP) assay-qPCR analysis in the region −2050/−50 of the *Gpx4* promoter after transfection with Jun plasmid or inducing by cholecystokinin (CCK, 5 μM) in 266-6 cells (*n* = 3). IgG, immunoglobulin G. Jun, Jun antibody. *, *p* < 0.05; **, *p* < 0.01; ***, *p* < 0.001; ns, not significant.

We first examined Jun-mediated GPX4 expression response in 266-6 cells, and immunoblotting assays showed that GPX4 expression was significantly reduced by Jun overexpression (Figure 3A). Several deletion promoter mutants of *Gpx4* were cloned into the luciferase reporter system (Luc1 to Luc3; Figure 3B). *Gpx4* activity was inhibited by Jun overexpression only in Luc1 constructs, indicating that the GPX4 promoter’s minimum Jun binding site was between 2050 and 1070 base pairs (bp). Using JASPAR promoter analysis methods, the potential binding motif sequences in the area were predicted. Thereafter, using the mutant *Gpx4* promoter sequence (ATGTCCTGAGTCACTCCTG→CGTGAAGTCTGACAGAAGT) at the Jun binding region (residues −1441 to −1423), we created a mutant luciferase reporter plasmid that we named *Gpx4* Jun-mut (Figure 3C). The control was a reporter system with a wild-type *Gpx4* promoter (*Gpx4* wt). When *Gpx4* wt reporter system was transfected into cells, overexpression of Jun inhibited *Gpx4* luciferase activity; however, this impact was reversed when the Jun binding site was altered (Figure 3D). We also conducted ChIP tests and found that the −1441 to −1423 bp region of the *Gpx4* promoter recruited Jun, this was induced by an overexpression of Jun or CCK (Figure 3E) in 266-6 cells. These results collectively demonstrated that the *Gpx4* promoter’s region between positions 1441 and 1423 is Jun’s functional binding site.

### 3.4. AP-1 Inhibitor Reversed GPX4 Suppression and Alleviated Ferroptosis in AP

**Figure 4:**
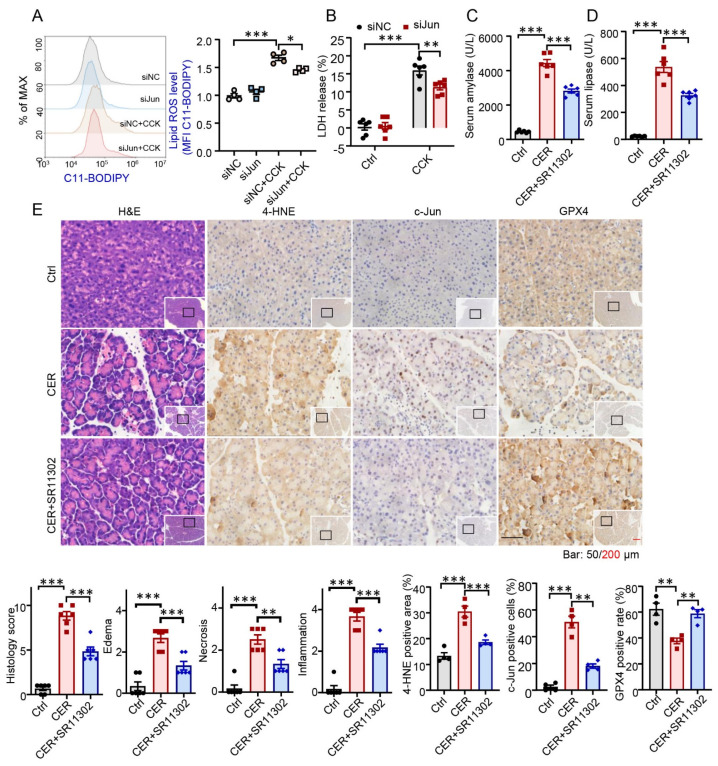
Inhibition of activating protein-1 (AP-1) reversed glutathione peroxidase 4 (GPX4) suppression and improved ferroptosis in acute pancreatitis. (**A**,**B**) After transfection with Jun siRNA for 36 h, cells were treated with cholecystokinin (CCK, 5 μM) for 6 h, and lipid reactive oxygen species (ROS) levels and cell death were determined (*n* = 4–6). (**C**,**D**) Serum amylase and lipase levels (*n* = 6). (**E**) Representative hematoxylin and eosin (H, E) staining of pancreatic tissues of vehicle cerulein-induced mice with or without SR11302 at magnifications of 100× and 400× (*n* = 6). Representative photomicrographs of pancreas stained for 4-hydroxynonenal (4-HNE), Jun, or GPX4 were assessed using immunohistochemistry (IHC) (*n* = 4).*, *p* < 0.05; **, *p* < 0.01; ***, *p* < 0.001; ns, not significant.

The goal was to investigate whether inhibition of AP-1 can protect AP injury via alleviating ferroptosis in vitro and in vivo. First, we detected AP-1-mediated oxidant response in CCK-induced 266-6 cells. Examination of C11-BODIPY 581/591 staining revealed that Jun knockdown significantly decreased lipid ROS levels (Figure 4A). Decreases of LDH levels were also observed in the siJun and CCK-treated groups (Figure 4B). Thereafter, we determined the effect of the AP-1 inhibitor, SR11302 [23], on ferroptosis and AP in vivo. SR11302 was intraperitoneally injected immediately after the first CER injection. Amylase and lipase levels in the serum were found to be significantly lower in the CER + SR11302 group than in the CER groups (Figure 4C,D). Consistent with the enzymatic indicators, SR11302 markedly reduced histological injury accompanied by decreased edema, inflammatory cell infiltration, and acinar cell necrosis (Figure 4E). Immunohistochemical staining assays of pancreatic tissues showed that 4-HNE and Jun were also inhibited by SR11302. The expression of GPX4 was significantly downregulated in CER-induced AP; however, it was significantly reversed by SR11302. Collectively, these results suggested that inhibition of AP-1 alleviates GPX4-dependent ferroptosis and decreases the severity of AP.

### 3.5. Gpx4 Deletion Abrogates the Anti-Ferroptosis and Anti-AP Effects of AP-1 Inhibitor

**Figure 5:**
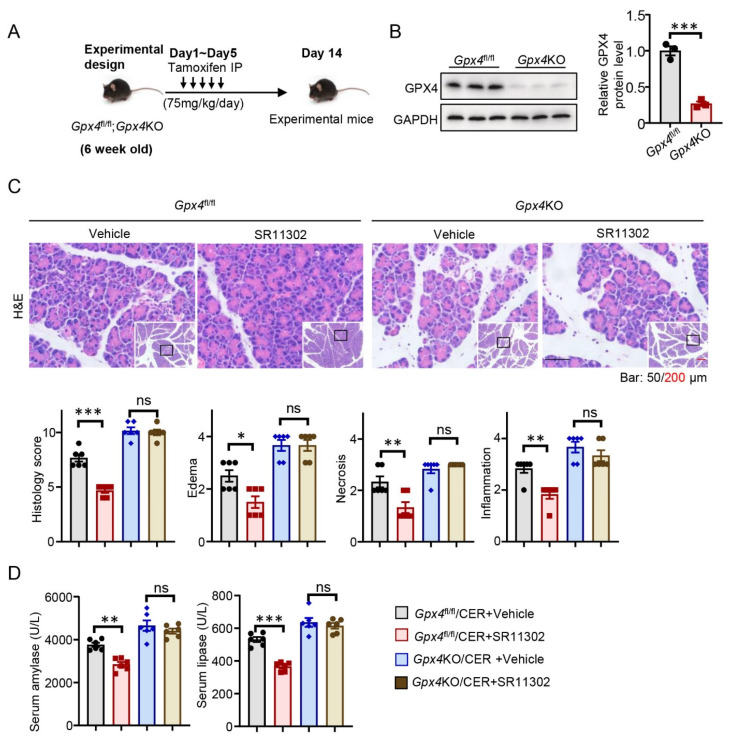
Glutathione peroxidase 4 (*Gpx4*) deletion abrogates the anti-ferroptosis and anti-acute-pancreatitis effects of activating protein-1 (AP-1) inhibitor. (**A**) The experimental design of *Gpx4*^fl/fl^ or *Gpx4*KO mice. (**B**) The protein level of GPX4 in pancreas of *Gpx4*^fl/fl^ or *Gpx4*KO mice (*n* = 3). (**C**) Representative hematoxylin and eosin (H,E) staining of pancreatic tissues of the cerulein (CER)-induced *Gpx4*^fl/fl^ and *Gpx4*KO mice with or without SR11302 in magnifications 100× and 400× (*n* = 6). (**D**) Serum amylase and lipase levels as indicated (*n* = 6). *, *p* < 0.05; **, *p* < 0.01; ***, *p* < 0.001; ns, not significant.

To learn more about the critical role of GPX4 in pancreatic injury pathogenesis, we first established a mouse model with the conditional KO of *Gpx4* in the pancreas of mice (*Ptf1a*^Cre^*Gpx4*^fl/fl^), as depicted in the schematic diagram (Figure 5A). GPX4 expression was also markedly repressed (Figure 5B). Thereafter, we used the AP-1 inhibitor, SR11302, for further experiments. SR11302 did not reduce the histological injury induced by CER in *Gpx4*KO mice (Figure 5C). The serum amylase and lipase levels did not change (Figure 5D). These results confirmed that inhibition of AP-1 by SR11302 reversed GPX4 reduction and ameliorated the pathology AP in a GPX4 dependent manner.

## 4. Discussion

There are several causes of pancreatitis, mainly including biliary pancreatitis, alcoholic pancreatitis, and hyperlipidemia [28]. Oxidative stress is considered a crucial mediator of early local events associated with AP and the associated progression of pancreatitis [20]. Experimental AP and clinical studies have confirmed that oxygen free radicals and lipid peroxide levels increase in the tissue and plasma, and these alterations are related to the severity of AP [29,30]. The collapse of the oxidation balance caused by the inactivation of GPX4 causes the membrane structure to be destroyed and accelerates the beginning of ferroptosis [15]. This study aimed to identify the mechanisms that regulate GPX4 expression. KEGG enrichment analysis of DEGs after CER-induced pancreatitis using GSE109227 showed that the differential genes were enriched in the ferroptosis pathway. GPX4 is an important lipid peroxidase in ferroptosis, and our study further revealed that using siRNA to specifically knockdown GPX4 in pancreas acinar 266-6 cells can produce high levels of lipid ROS and increase cell death. Our results further validate previously reported results [12,18,20,21,22]. Moreover, RNA-seq data revealed that CER-induced acinar ferroptosis showed a marked elevation of the transcription factor AP-1 protein Jun.

We used the lipid peroxidation inhibitor liproxstatin-1 to prove that inhibiting ferroptosis suppresses pancreatitis and AP-1 levels. A complex system of transcription factors that direct the expression of the genome by recognizing specific DNA sequences and play a role in various diseases [31]. AP-1 has tissue specificity and is pro-inflammatory in the pancreas [32,33]. We found that the expression of the AP-1 transcription factor was significantly upregulated after ferroptotic death in acute pancreatitis mouse models. As a transcriptional regulator, AP-1 can positively and negatively regulate gene expression [34]. We further found that AP-1 can bind to the promoter region of GPX4 to exercise transcriptional inhibition and that overexpression of AP-1 can reduce the expression level of GPX4. However, the interaction between AP-1 and GPX4 in patients with pancreatitis is unknown and requires further research. siRNA technology found that after the expression of Jun, levels of lipid peroxidation and the mortality rate caused by CCK decreased. Animal experiments with the AP-1 inhibitor SR11302 showed that inhibition of AP-1 can increase the expression of GPX4, reduce the level of lipid peroxidation, and significantly improve pancreatitis. Further investigations found that inhibiting AP-1 did not reduce GPX4 deficiencies-induced ferroptosis and mitigated acute pancreatitis. The activation of AP-1 plays an important role in ethanol toxicity and taurocholate-induced pancreatitis [35,36], and whether the AP-1/GPX4 regulatory axis plays an important role requires further study.

Studies have shown that the inflammatory response activates the coagulation cascade, which in turn promotes inflammation [37,38]. The activation of coagulation has been observed in both clinical and experimental AP, and the degree of activation was positively corelated with the severity of AP [39,40,41]. Further, the administration of anticoagulants, such as heparin [42,43,44], acenocoumarol (vitamin K antagonist) [45,46], and warfarin [47,48] exhibited protective and therapeutic effects in AP. Recent studies have also revealed a close association of coagulation with ferroptosis [49,50]. Tuo et al. [49] found that thrombin served as a potent ferroptosis inducer, which increased the expression of oncostatin M (an inflammatory cytokine) via AP-1 activation in macrophages [51], indicating that AP-1 may aggravate AP by participating in coagulation-associated ferroptosis pathway. It is worth noting that the reduced forms of vitamin K significantly inhibited ferroptosis [52,53], suggesting that coumarin as a vitamin K antagonist might promote ferroptosis by decreasing the reduced forms of vitamin K. This assumption is seemly contradictory to the anti-ferroptosis property of anticoagulants. Apparently, the roles of coagulation during ferroptosis and AP-1 in coagulation-associated ferroptosis need further investigation.

## 5. Conclusions

In summary, our results showed that AP-1 elevation aggravates ferroptosis and AP via the transcriptional inhibition of GPX4. Our research deepens the understanding of the regulation of ferroptosis in AP and provides a potential therapeutic target for AP prevention and treatment.

## Data Availability

Data are contained within the article.
